# Impacts on terrestrial biodiversity of moving from a 2°C to a 1.5°C target

**DOI:** 10.1098/rsta.2016.0456

**Published:** 2018-04-02

**Authors:** Pete Smith, Jeff Price, Amy Molotoks, Rachel Warren, Yadvinder Malhi

**Affiliations:** 1 Institute of Biological and Environmental Sciences, and ClimateXChange, University of Aberdeen, 23 St Machar Drive, Aberdeen AB24 3UU, UK; 2 Tyndall centre, School of Environmental Sciences, University of East Anglia, Norwich Research Park, Norwich NR4 7TJ, UK; 3 Environmental Change Institute, School of Geography and the Environment, University of Oxford, South Parks Road, Oxford OX1 3QY, UK

**Keywords:** biodiversity, climate change targets, land, greenhouse gas removal

## Abstract

We applied a recently developed tool to examine the reduction in climate risk to biodiversity in moving from a 2°C to a 1.5°C target. We then reviewed the recent literature examining the impact of (a) land-based mitigation options and (b) land-based greenhouse gas removal options on biodiversity. We show that holding warming to 1.5°C versus 2°C can significantly reduce the number of species facing a potential loss of 50% of their climatic range. Further, there would be an increase of 5.5–14% of the globe that could potentially act as climatic refugia for plants and animals, an area equivalent to the current global protected area network. Efforts to meet the 1.5°C target through mitigation could largely be consistent with biodiversity protection/enhancement. For impacts of land-based greenhouse gas removal technologies on biodiversity, some (e.g. soil carbon sequestration) could be neutral or positive, others (e.g. bioenergy with carbon capture and storage) are likely to lead to conflicts, while still others (e.g. afforestation/reforestation) are context-specific, when applied at scales necessary for meaningful greenhouse gas removal. Additional effort to meet the 1.5°C target presents some risks, particularly if inappropriately managed, but it also presents opportunities.

This article is part of the theme issue ‘The Paris Agreement: understanding the physical and social challenges for a warming world of 1.5°C above pre-industrial levels'.

## 1. Introduction

Perhaps the most remarkable outcome of the Paris Climate Agreement was a renewed focus on the aim of limiting global average temperature increase to ‘well below 2°C above pre-industrial levels’, and ‘pursuing efforts’ to limit it to 1.5°C. This has stimulated a new focus on understanding the challenges of achieving this target, but also differential costs and benefits of such ambitious climate targets. It is clear that immediate and aggressive mitigation action in all sectors is necessary to meet the 2°C target, with even this target probably requiring some atmospheric greenhouse gas removal during this century [1–3]. For the 1.5°C target, additional atmospheric greenhouse gas removal is even more likely to be required, and in greater quantity, to supplement immediate and aggressive mitigation [3].

Biodiversity is known to be sensitive to a range of global change drivers [4], including climate [5], land-use change [6] and land management [7]. Widespread biodiversity loss would be expected to significantly reduce ecosystem services globally, because even small declines in species populations can negatively impact service provision [8], and such loss of function in turn threatens human well-being [9]. Climate impacts may have differential direct impacts on biodiversity at 1.5°C and 2°C, but striving to achieve the 1.5°C target might also have indirect impacts on biodiversity via changes in land use and management implemented to help reach the 1.5°C target.

In this paper, we examine both direct impacts and indirect impacts of striving to reach the 1.5°C target. We examine (§2) the difference in risks to biodiversity of a 1.5°C world, relative to a 2°C world, and (§3) impacts on biodiversity of land-based efforts to reach the 1.5°C target. In the final section, we discuss the relative importance of direct and indirect impacts of the 1.5°C target on biodiversity, and we highlight some exciting current developments in this field and propose areas for future research. Owing to limitations in the datasets available, and our focus on climate envelopes, we focus our analysis on land regions, though we know that one of the most important reductions of risk to biodiversity under a 1.5°C target may be found in the marine biosphere, in particular through reduced levels of ocean acidification rather than through climate. The benefits to terrestrial biodiversity discussed here build on the previously established benefits for ocean biodiversity, which are not further discussed in this paper.

## 2. Difference in impacts on biodiversity of a 1.5°C world relative to a 2°C world

In this section, we examine the difference in risks to biodiversity of a 1.5°C world relative to a 2°C world incurred as a result of direct climate change impacts. Climate change poses risks to biodiversity, globally, regionally and locally [10,11]. Changes in phenology [12,13], species' ranges [14,15], ecological interactions [16] and primary productivity [17] have already been observed, and many studies have attributed such changes (as a whole or in part) to anthropogenic climate change (see [18] for a synthesis). Species face a challenge in being able to track their preferred climate space across what is often an increasingly fragmented landscape [17], in terms of both the speed of movement required to adapt and obstacles to movement. Furthermore, some of these barriers to movement are likely to increase in the future (see §3). Many studies have examined potential future impacts of climate change on biodiversity using a variety of modelling techniques. This includes models showing the potential for losses of range exceeding 50% across large fractions of species globally or regionally due to climate change (e.g. among 50 000 species studied, 57 ± 5% of plants and 34 ± 7% of animals were projected to lose over half their climatic range for a warming of approximately 3.6°C above pre-industrial levels [19]). Other studies have looked specifically at the potential for increasing risks of extinction at higher warming levels. A meta-analysis suggested 20–30% of plant and animal species would be at risk of extinction if global mean temperatures exceeded a warming of 2–3°C above pre-industrial levels [14], with another study predicting 32–34% of Western Hemisphere birds with increased risks of extinction for warming of approximately 3°C above pre-industrial levels [20]. Traits-based analyses have also produced similarly large estimates of the proportions of coral, bird and amphibian species with increased extinction risks for warming of approximately 3°C above pre-industrial levels [21]. Even for mid-range warming of 1.8–2°C, studies have shown predictions of 15–37% of endemic species being ‘committed to extinction’, demonstrating the potential severity of even moderate levels of climate change [22].

All such studies are couched in uncertainties. There remains uncertainty and debate about the plasticity of species' physiologies and ability to maintain or newly construct biotic interactions under climate change, and the relationship between ‘commitment to extinction’ and actual extinction rates. Nevertheless, such exercises are valuable in highlighting regions and taxa of high relative risk and which may be foci of conservation or climate adaptation actions.

While many studies have specifically looked at climate impacts on biodiversity at higher temperatures, or over different future time periods (see [23] for a meta-analysis), few have specifically looked at the benefits of climate mitigation with respect to potential impacts on biodiversity [19,24]. The Wallace Initiative is a global effort to model the potential impacts of climate change on biodiversity, to identify areas of climatic refugia for biodiversity and to analyse the potential impacts on protected areas worldwide. The first analyses with these data specifically looked at the proportion of species that are projected to lose over 50% of their bioclimatic range (at a spatial resolution of approx. 50 km × 50 km) due to climate change alone, calculated separately for plants and animals, for seven climate models (CMIP3) and a range of emission scenarios [19]. In this study, limiting warming to 2°C as opposed to 4°C was found to reduce the proportion of animals losing over half their climatic range from 42% (±7%) to 12% (±3%), and plants from 57% (±6%) to 23% (±4%) of the 50 000 species studied globally.

It should be noted that projection of range shifts of species in the lowland tropics (the location with most present biodiversity and projected biodiversity loss) carries particularly high uncertainty. This is because climate warming carries many tropical regions into novel climates [25], i.e. warmer climates not currently experienced on Earth. Hence the upper temperature range of species ranges is defined by currently observed temperatures, rather than by observed geographical ranges, without clear biogeographical insight into whether species will be able to adapt to temperatures a few degrees warmer. This necessarily causes massive decline in diversity in the lowland humid tropics (e.g. Amazonia or Central Africa [26]). There is substantial uncertainty as to whether most tropical organisms will be able to cope with a few degrees of warming. On the one hand, many tropical species may be able to cope with slightly higher temperatures because of rapid adaptation or generational turnover, or because there is no biotic pressure from in-migrating species adapted to higher temperatures [27]. On the other hand, other strands of evidence suggest that tropical organisms may have physiologies uniquely sensitive to small net increases in temperature, because of unusually low seasonal and inter-annual variability in temperature [28]. Some taxa such as plants may be more plastic in their physiology than others such as arthropods. This scientific uncertainty means that predictions of large-scale loss of biodiversity in the tropics under moderate warming should be treated with caution. Despite this uncertainty, and with these caveats in mind, it is still useful for such biogeographical analyses to highlight potentially sensitive and resilient regions, and in particular to examine how risk shifts between different climate scenarios.

In this paper, we apply a similar tool to plant [24] to examine the question of how much reduction there is in climate risk to Warren *et al.* biodiversity in moving from a 2°C to a 1.5°C target, and then examine regional differences in the relative impacts. In so doing, we follow the procedure of Warren *et al.* [19] by, respectively, including or excluding an ability for species to disperse in order to track shifting climates across the Earth's surface at rates informed by the literature (for further detail see [19]).

### (a) Global benefits to reducing potential impacts on biodiversity at 1.5°C versus 2°C

We reanalysed the original Wallace Initiative data [19] to look at the potential benefits of constraining global mean temperature rise to 1.5°C versus 2°C. While pathways to 1.5°C were not specifically examined for this project, the potential impacts on biodiversity at 1.5°C can be estimated from the time-series scenarios examined. The main difference from looking at specific pathways, versus the approach presented here, comes in the overall potential for species (primarily birds and mammals) to move to keep up (or catch up) with climate change and colonize new territories. This is dependent on not only the pathway to reaching 1.5°C (whether it is slow and gradual, quick with a long plateau, or an overshoot with a decline to 1.5°C) but also whether movement pathways are blocked by barriers, especially in the form of incompatible land uses. For this reason we are only presenting a preliminary estimate of the results for the no-dispersal scenario for plants, which disperse in general only slowly, in this paper.

While a temperature difference of 0.5°C may seem small, the potential global biodiversity risks avoided by constraining warming to 1.5°C rather than 2°C above pre-industrial levels may be significant. At 2°C the models project that around 23% (±4%) of plants would lose more than half of their climatic range, while at 1.5°C risks to plants could be reduced to an estimated 7–13%. Thus, risks from climate change to plants (in terms of loss of climatic range) are estimated to be significantly reduced at 1.5°C versus 2°C (estimated from Warren *et al*. [19], no-dispersal scenario). Similar reductions might be expected for animals. Further, there would be an increase of 5.5–14% of the globe that could potentially act as climatic refugia for plants and animals, which is an area equivalent to the current global protected area network. These benefits would be reduced or even negated if 1.5°C was reached in an overshoot scenario [3], depending on the length of time and temperature reached during the overshoot.

### (b) Regional differences

**Figure 1.**
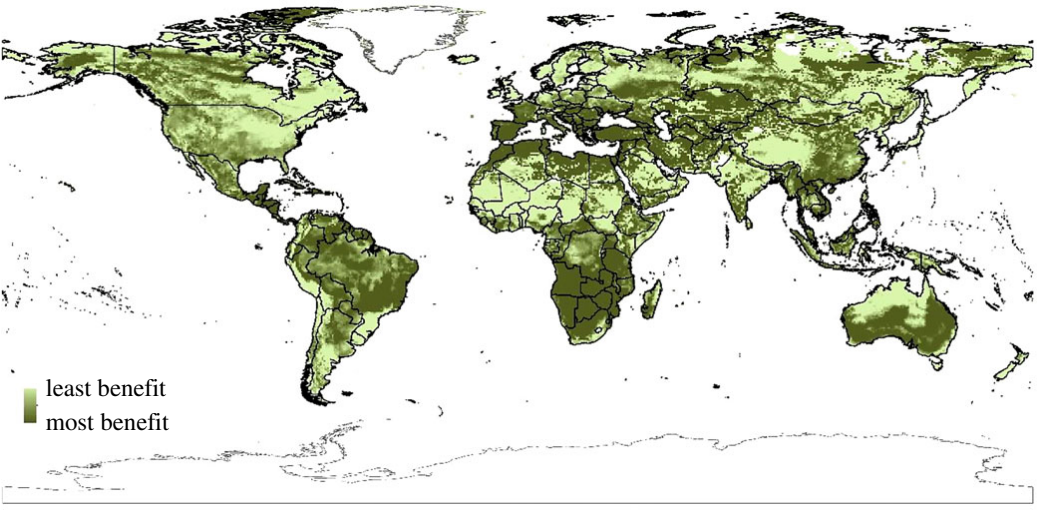
The areas showing the greatest benefit to maintaining plant species richness at 1.5°C versus 2°C. The darker the green, the greater the benefit in mean (out of seven models) species richness preserved. Areas that are lighter green show the same approximate numbers of species at 1.5°C and 2°C [19]. Full details of the methods used can be found in Warren *et al*. [19].

The benefits of mitigation to biodiversity conservation are not evenly distributed across the globe. An example of this, comparing 4°C versus 2.5°C, can be seen in Warren *et al.* [19], fig. 3. Furthermore, while constraining warming to 1.5° versus 2°C will have global benefits, some regions will continue to benefit more than others. Figure 1 shows regions with the greatest reduction in potential species richness loss for plants at 1.5°C versus 2°C.

The approach described in this section provides an assessment of possible changes of moving from a 2°C to a 1.5°C target, but it should be noted that there are many metrics of local and regional change, and selecting different metrics can yield different outcomes. Garcia *et al*. [29], for example, compared six metrics of change at local (climate anomalies, climate extremes, and changes in seasonality) and regional (change in area of analogous climate, novel climates, and change in distance to analogous climates) level, and found that they provide different and complementary information. Despite the variety of different approaches available, and the presentation of only one such method in this section, the findings outlined here are indicative of potential changes, suggesting that further detailed study is required to assess fully the implications for biodiversity of moving from a 2°C to a 1.5°C target.

Having reviewed the risks to biodiversity of a 1.5°C world relative to a 2°C world incurred as a result of direct climate change impacts in this section, in §3 we examine the effects on biodiversity of land-based efforts to meet the 1.5°C target.

## 3. Impacts on biodiversity of land-based efforts to reach the 1.5°C target

In addition to the direct differential impacts upon biodiversity of 1.5°C versus 2°C warming described in §2, there are likely to also be indirect impacts, driven by efforts to achieve the 1.5°C target. These biodiversity impacts could be driven by land-use change or land-management change through, for example, changes in agricultural management aiming to deliver greenhouse gas mitigation [30], or by land-use change arising from implementation of land-based greenhouse gas removal (sometimes referred to as negative emissions) technologies [1,31,32]. In this section, we examine the evidence for potential impacts on biodiversity, of land-use and land-management change, driven by efforts to achieve the 1.5°C target through land-based options. While it is difficult to quantify in absolute terms the difference in impact between a 1.5°C versus a 2°C target, the higher level of mitigation or greenhouse gas removal ambition required to achieve 1.5°C would necessitate that such actions be applied more aggressively and more widely. We present the likely impacts of these actions on biodiversity, and assess the implications of their more aggressive/widespread application to meet a 1.5°C target.

### (a) Impacts on biodiversity of additional climate mitigation in the agriculture, forestry and other land-use sector to reach the 1.5°C target

Climate mitigation in the agriculture, forestry and other land-use (AFOLU) sector is implemented via change in management of land, or via land-use change [33]. Land-use change options that provide mitigation benefits include the restoration of farmed organic peatlands, restoration of degraded land [34], afforestation (though we discuss this in §3b) [33], and the protection of large carbon stocks through, for example, wetland/peatland protection [35] or reduced deforestation and degradation [36]. Most of these land-use change activities are likely to provide improvements for biodiversity status [37], because they all involve de-intensification of land use and/or reversion to a state closer to the undisturbed ecosystem, which is a positive outcome for most biodiversity indicators. Proper planning, tied with the findings from §2, would actually provide adaptation benefits concomitant with mitigation. Most mitigation in the AFOLU sector, however, is realized through land-management change, i.e. the land use remains the same (e.g. cropland, grazing land, forest), but the management interventions are modified to reduce greenhouse gas emissions.

On croplands, mitigation practices include improved rotations with the greater use of cover crops and nitrogen-fixing crops, reducing the intensity of tillage, improved residue management, optimized fertilization (correct amount, placement and timing), the better use of organic amendments such as manure and straw, and optimized water management (particularly for rice). These are described and reviewed by Smith *et al*. [33,34]. Of these practices, reducing the use of mineral fertilizer could have beneficial impacts on some microbial components of biodiversity, with, for example, methane oxidizers known to be suppressed by large quantities of nitrogen fertilizer [38]. Over-fertilization (mineral or organic) is known to have significant off-site effects, particularly through the eutrophication of watercourses and water bodies [39], so optimization of fertilizer use as a climate mitigation measure is likely to have positive impacts on biodiversity locally, but also off-site regionally (within the same watershed/catchment [39]), and over larger scales, because atmospheric N deposition originating from agriculture adversely affects biodiversity across terrestrial biomes [40]. There may, however, be trade-offs between yield and biodiversity if fertilizer applications are reduced below plant requirements.

Improved, diversified rotations, including shorter periods where the ground is left bare, are also likely to improve soil biodiversity, because plant cover is provided all year round and carbon inputs will be higher [41]. The exact impact of the improved rotations will depend upon the crops included in the rotation and how they are grown (grown in sequence/under-sown, etc.), but relative to a rotation where the ground is bare for significant parts of the year, the biodiversity impact is expected to be positive. Given that plant cover is known to reduce soil loss through water and wind erosion [42], and that large deposits of eroded soil are known to increase turbidity [43] and disadvantage biodiverse watercourses [44] and coastal waters (e.g. [45]), improved rotations might also be expected to benefit biodiversity at the watershed/catchment scale or at coastal margins. Management systems emphasizing crop diversity can also reduce insect pests and provide refuge for natural enemies and pollinators, especially if mass-flowering crops are used in rotations [46].

As with improved rotations, reduced tillage intensity, particularly when combined with improved residue management (leaving crop residues on the field), is likely to reduce erosion [47], so the local and watershed/catchment-scale benefits will be similar. Reduced tillage can also increase soil organic matter, which increases soil quality through improved structure and increases soil microbial activity, which is negatively influenced by soil tillage [48]. Additional local soil biodiversity benefits might also be realized through greater number and diversity of earthworms and mesofauna, which are known to be more prevalent under reduced tillage systems compared to conventionally tilled soils [49]. One indirect impact might arise from reduced tillage if additional herbicide is required to reduce weeds as a result of reduced mechanical weeding by ploughing [50], which would be likely to have a negative impact on plant and insect biodiversity.

On grazing lands, mitigation practices include manipulation of grazing intensity, optimized fertilization (correct amount, placement and timing), the better use of organic amendments such as manure, fire management, use of deeper-rooting species and increased legume shares in the sward (reviewed in [33,34]). Reduction of over-fertilization on grazing lands (and replacing external nitrogen inputs by increasing legume shares) will have similar beneficial impacts as for croplands described above, providing both local and watershed/catchment-scale benefits for biodiversity. Rotational grazing can also more effectively imitate natural feeding patterns of migratory herbivores, resulting in increased plant biodiversity and improved soil quality [51]. The relationship between grazing intensity and biodiversity is complex, but overgrazed systems lose biodiversity relative to optimally grazed grasslands [52], and for some grasslands, highly valued biodiversity is maintained by moderate grazing [52]. If grazing intensity is optimized, it is therefore likely to improve biodiversity relative to un-optimized practice. Overgrazing can also lead to bare soil, which will increase erosion risk [53]—so impacts on reduced erosion through optimal grazing will be similar to those described for croplands above. Deeper-rooting species might also reduce erosion risk and the concomitant impacts on local and watershed/catchment-scale biodiversity. The impact of fire management on biodiversity is also complex. In some systems, frequent fire prevents woody encroachment and maintains biodiverse grassland ecosystems [54], though there is also evidence that fire suppression can increase fuel load, so that when fire does occur, the fire burns more intensely (though see [55]). This can have a negative impact on biodiversity by changing the community structure, increasing the risk of extinction. Furthermore, increasing levels of CO_2_ are leading to woody plants in grasslands becoming more fire-resistant. Burning can also accelerate soil erosion [53], with the negative biodiversity consequences noted above.

Forestry measures for climate mitigation include reduced deforestation and degradation, and improved forest management. Reduced deforestation and degradation are likely to benefit biodiversity in that they preserve habitat in a condition closer to natural [36,56]. The potential benefits of reduced deforestation and degradation to biodiversity have been mapped, although these findings do not take into account any changes due to climate change [57]. Improved forest management includes practices such as reduced impact logging [58]. Such practices are also likely to have less impact on biodiversity than clear-fell [59], and also leave more necromass in the forest, which is important for forestry food webs and biogeochemical cycling.

In addition to the practice-specific impacts described above, there are also systemic changes to food/fibre production that have been proposed as climate mitigation measures. These include sustainable intensification [60,61], whereby environmental impact is reduced relative to the unit of product. In greenhouse gas terms, this is expressed through decreased emission intensity (GHG emitted per unit of product) [33]. By targeting areas of low efficiency, environmental impacts of intensive agriculture could be limited and the need for agricultural expansion would be reduced [62]. For this intensification to be sustainable, by definition, it must have no adverse effects on biodiversity. Sustainable intensification, then, would at worst be neutral in terms of biodiversity on the land on which it is practised, and could potentially spare land for other uses if food production can be concentrated on a smaller land area [63–66]. Closing yield gaps through sustainable intensification would decrease global cropland and grassland areas required for food production by 37% and 10%, respectively [66], relative to current yield trends without yield gap closure, while adding an estimated 2.3 billion tonnes of new production—a 58% increase [62]. However, closing the yield gap without environmental degradation will require new approaches, including the reform of conventional agriculture, adopting lessons from organic systems and precision agriculture as well as overcoming challenges such as distribution of inputs and seed varieties [62]. Other systemic changes in the food system with considerable mitigation potential are dietary changes (largely via reduction in livestock product consumption [67]) and waste reduction [66]. Both of these demand-side measures also reduce the pressure on land [30,68,69]. Combining dietary change (to healthy global diets) and waste reduction by 50% could reduce global cropland and grassland areas by 17% and 36%, respectively, compared to yield gap closure alone [68]. This creates the opportunity for land sparing [66], potentially providing considerable benefits for wildlife conservation and biodiversity [66], though there could be rebound effects, as the land spared does not necessarily match with priority areas for biodiversity conservation.

For all of the measures discussed in this section, it is possible that all might be used in efforts to meet the 2°C target, which could mean little difference to biodiversity. However, given the known challenges of meeting a 1.5°C target, we might expect efforts to reach 1.5°C to lead to them being applied to their maximum extent and over large geographical areas, with the impacts on biodiversity discussed in this section thereby accentuated. Table 1 summarizes the likely biodiversity impact associated with more aggressive/widespread implementation of the mitigation measures discussed in this section.

**Table 1.** Summary of the likely biodiversity impacts associated with more aggressive/widespread implementation of land-based climate change mitigation measures.

mitigation type	mitigation measure	impact on processes affecting biodiversity	local biodiversity impact	catchment-scale biodiversity impact	regional/global biodiversity impact
land-use change	restoration of farmed organic peatlands	peatland restoration/rewetting	+	+	+
land-use change	restoration of degraded land	soil/vegetation cover restored	+	+	+
land-use change	protection of large carbon stocks (peatlands/reduced deforestation and degradation)	peatland/forest protection	+	+	+
cropland management	improved rotations	reduced soil erosion risk—reduced turbidity		+	+
cropland management	reduced tillage intensity	reduced impact on earthworms and mesofauna; requirement for additional herbicides; reduced soil erosion risk—reduced turbidity	+/−	+/−	+/−
cropland management	improved residue management	reduced soil erosion risk—reduced turbidity		+	+
cropland management	optimized fertilization	reduced adverse impact on soil microbiota; reduced nutrient loss reducing eutrophication; reduced N deposition	+	+	+
cropland management	better use of organic amendments	reduced nutrient loss reducing eutrophication		+	+
grazing land management	manipulation of grazing intensity	reducing overgrazing; greater plant production and less bare soil; reduced soil erosion risk—reduced turbidity		+	+
grazing land management	optimized fertilization	reduced adverse impact on soil microbiota; reduced nutrient loss reducing eutrophication	+	+	+
grazing land management	better use of organic amendments	reduced nutrient loss reducing eutrophication		+	+
grazing land management	fire management	prevention of woody encroachment; fewer but more intense fires	+/−	+/−	+/−
grazing land management	deeper-rooting species	reduced soil erosion risk—reduced turbidity		+	+
forest management	REDD+	reduced deforestation and degradation	+	+	+
forest management	selective/low-impact logging	less impact than clear-fell, more necromass; reduced erosion losses—reduced turbidity	+	+	+
food system change	sustainable intensification	concentrated production on smaller land area	+/−	+/−	+
food system change	dietary change	fewer livestock products in the diet—more efficient production – less pressure on land—land sparing	+	+	+
food system change	food waste reduction	less pressure on land—land sparing	+	+	+

With a few exceptions, the majority of mitigation measures summarized in table 1 confer biodiversity benefits, either locally, at catchment scale or globally. Global impacts arise because local impacts are spread over large areas when there is widespread application of practices (e.g. by improved cropland or grassland management over the 40–50% of the global land surface used for agriculture), or because the impacts of the mitigation measure can occur in distant locations (e.g. eutrophication of distant water bodies or N deposition from changed fertilizer management [40]). The implementation of these measures in an attempt to meet the 1.5°C target would, therefore, largely be consistent with biodiversity protection, though governance will be critical to achieving mitigation and biodiversity conservation goals [36,70].

### (b) Impacts on biodiversity of implementation of land-based greenhouse gas removal technologies to reach the 1.5°C target

Terrestrial greenhouse gas removal technologies [1,2,32] include: (1) bioenergy with carbon capture and storage (BECCS [71]); (2) direct air capture of CO_2_ from ambient air by engineered chemical reactions [72,73]; (3) enhanced weathering of minerals [74–76], where natural weathering to remove CO_2_ from the atmosphere is accelerated, and the products are stored in soils, or buried in land/deep ocean; (4) afforestation and reforestation [70,77,78] to fix atmospheric carbon in biomass and soils; (5) soil carbon sequestration through changed agricultural practices (which include activities such as less invasive tillage with residue management, organic amendment, improved rotations/deeper-rooting cultivars, optimized stocking density, fire management, optimized nutrient management and restoration of degraded lands) [32,34]; and (6) converting biomass to recalcitrant biochar, for use as a soil amendment [32,79]. There will be an interaction between the use of greenhouse gas removal technologies and the direct impacts described in §2, because greenhouse gas emissions are used to manage an overshoot of emissions/temperature increase. Therefore, scenarios that employ greenhouse gas removal to manage overshoot are likely to result in additional direct impacts on biodiversity compared to those that do not result in overshoot, because temperature will be higher than 1.5°C for a period of time [3]. These issues will be examined in the forthcoming Intergovernmental Panel on Climate Change Special Report on meeting the 1.5°C target.

Of these options, some have an obvious land footprint and, when implemented, prevent the land being used for other purposes (e.g. afforestation and reforestation, BECCS, the mineral mining components of enhanced weathering), while others use land but do not prevent it from being used for its current purpose (e.g. soil carbon sequestration, biochar, the land spreading component of enhanced weathering), and still others may have an indirect footprint (a direct capture plant has a small land footprint, but if powered by renewable energy such as wind or solar, the indirect land footprint could be very large [80]). Table 2 presents the land footprint of greenhouse gas removal technologies, expressed per tonne of CO_2_ carbon removed from the atmosphere, with notes on whether or not the land can be used for other purposes when the technology/practice is implemented on it [1,32,81].

**Table 2.** Land footprint of greenhouse gas removal technologies, expressed per tonne of CO_2_ carbon removed from the atmosphere (data from [1,32,79] and references therein).

	GHG removal rate per unit land		land area per unit of GHG removal		
	low	high	low	high	
technology	t-Ceq./ha	t-Ceq./ha	ha/t-Ceq.	ha/t-Ceq.	current land use still possible?
BECCS	3	12	0.1	0.4	no
afforestation/reforestation	3.4	3.4	0.1	0.6	no
soil carbon sequestration	0.03	1	1	33	yes
biochar	1.15	7.5	0.13	0.87	yes
direct air capture	1818	1818	0.001	0.001	no
enhanced mineral weathering	0.82	10.91	1.22	0.09	no/yes^a^

^a^No for mineral mining sites; yes for land upon which the ground rock is spread.

The land footprint of direct air capture technologies is small, so the direct impact on biodiversity is also likely to be small. The land area required for mining minerals for enhanced weathering is small globally, but mining has devastating local biodiversity impacts where it occurs due to habitat destruction unless ameliorated, though site restoration is possible after mining ceases [82]. For land upon which the crushed/ground mineral is spread, current activity on the land could continue if rates are relatively low. When applied at low rates of 10 t rock ha^−1^ yr^−1^ [76], agricultural/forestry use of the land could continue, though these rates are still higher than those regularly used in agriculture for liming [83] and could have negative impacts on biodiversity. However, high application rates of 50 t rock ha^−1^ yr^−1^ [76] are incompatible with agriculture, so only low rates would not lead to pressure on land, and thereby minimize biodiversity impacts. Soil carbon sequestration is a headline indicator for soil quality [84] and soil health [85], and hence is likely to support local biodiversity. If soil carbon sequestration is used to help restore degraded lands, global biodiversity benefits could follow. Biochar could have negative impacts on soil biota if contaminated, but when created from clean feedstock, it is generally regarded as beneficial to soil microbiota by providing microsites for colonization [86]. Soil carbon sequestration and biochar addition, when practised sustainably, would, therefore, have neutral to positive impacts on biodiversity.

BECCS and afforestation/reforestation are the greenhouse gas removal technologies that occupy land so that it cannot be used for other purposes, though some forms of afforestation, e.g. agroforestry, still allow agricultural production [87]. Vast land areas could be used for greenhouse gas removal if greenhouse gas removal on the scale of approximately 10 Gt CO_2_ is necessary [1]. Such levels of implementation would require land areas of 380–700 Mha for BECCS and 970 Mha for afforestation/reforestation [1]. To put these figures in context, the total agricultural land area in 2000 was approximately 4960 Mha, with an area of arable and permanent crops of approximately 1520 Mha [88], so the area for BECCS represents 7–25% of agricultural land, and 25–46% of arable plus permanent crop area. Afforestation at a level to deliver equivalent greenhouse gas removal (970 Mha) represents 20% of the total agricultural land, and 64% of arable plus permanent crop area [1]. For both BECCS and afforestation/reforestation, then, the land footprint, and therefore the biodiversity impact, could be very large—unless afforestation is implemented in a way that supports biodiversity [89].

**Figure 2.**
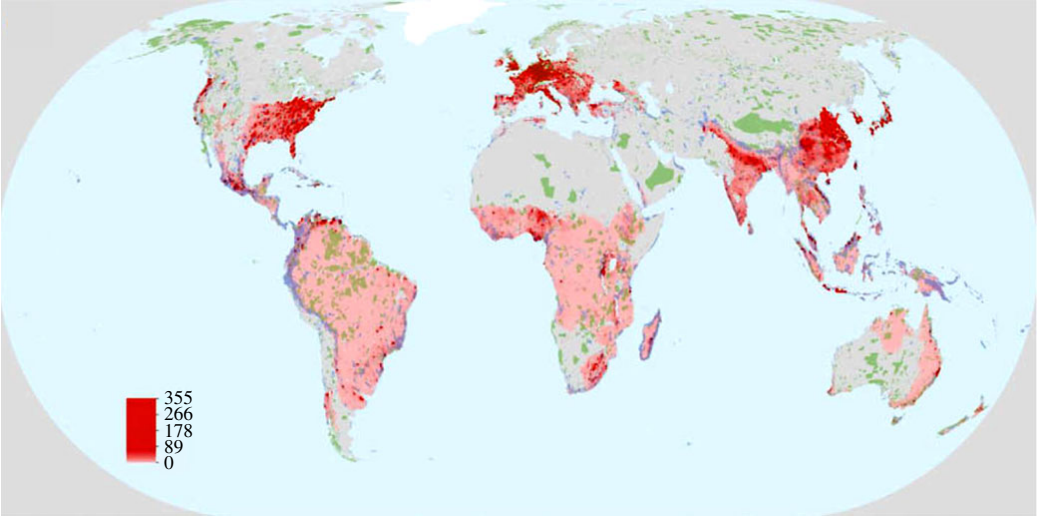
Overlap between power generation potential (GJ ha^−1^ yr^−1^; red colour gradient; see legend) for bioenergy (here represented by *Miscanthus* × *giganteus* as simulated by the MiscanFor model), constrained by energy demand, costs and carbon, overlaid with current protected areas (green shading) and global top 17% areas for protected area expansion (blue shading). Areas with no power generation potential are in grey. For bioenergy, no data were available for Greenland. (Reproduced with permission from Pedroli *et al*. [91].) Full details of the methods used can be found in Pedroli *et al*. [91].

For BECCS, though some bioenergy crops have been shown to provide biodiversity benefits when grown on former cropland [90], very large-scale deployment is often suggested to be associated with potential biodiversity loss [91], either directly, or indirectly by displacing food production to other areas where it replaces natural ecosystems [92]. Santangeli *et al.* [93] recently examined the potential conflicts between renewable energy production and biodiversity by examining overlap between energy production potential and current and future protected areas, with bioenergy showing the largest potential conflicts with biodiversity (e.g. figure 2). If biomass is extracted from existing forests for BECCS, fears have been expressed that this could lead to forest degradation [94], with concomitant loss of biodiversity. The areas identified as having the highest potential BECCS, in terms of biomass production potential from dedicated energy crops, in figure 2 (showing *Miscanthus* as the example bioenergy crop) can be compared with the areas reserved for biodiversity benefits in figure 1. The overlap and potential impacts on biodiversity are high for plants in the United States, Central America, West and South Africa, United Kingdom, India and Indonesia. For animals, the degree of overlap between refugia at 2°C and power generation potential is high, with most areas that have been identified as having high power generation potential also being climatic refugia for most animals. Thus, land-use changes on the order of that required by BECCS to meet a 1.5°C target (380–700 Mha [1]) would have potentially significant impacts on biodiversity.

For afforestation/reforestation, biodiversity impacts will depend upon how afforestation occurs (species planted, method of establishment) and the previous use of the afforested land. Although, in most cases, afforestation would benefit biodiversity, in some cases it could have negative impacts on native flora and fauna; for example, if based on tree plantations, naturally open habitats would be replaced by low-biodiversity ecosystems with a high water uptake [95]. Biodiversity is known to be lower in production forestry relative to natural forest, but can be managed [96]. If monoculture production forestry is used to maximize carbon dioxide removal, biodiversity could suffer, but if biodiverse mixed forestry is used, biodiversity could be enhanced by afforestation/reforestation. There is likely to be a trade-off between maximizing greenhouse gas removals and providing a wider range of ecosystem services, including biodiversity [96]. Previous land use also plays a role; the widespread planting of production forestry on UK peatlands during the 1940s–1980s is widely regarded as a conservation disaster [97], and planting on deep peats has since been discontinued [98]. Planting of biodiverse mixed woodland on degraded former cropland, however, is likely to enhance biodiversity, can even be inserted in agricultural landscapes [99] and might act as an adaptation in providing connectivity between habitats to potentially allow some taxa to shift with climatic changes. The impacts of afforestation/reforestation on biodiversity is therefore quite context-specific, but could usually be implemented in a way that enhances biodiversity [89].

At the planetary level, greenhouse gas removal technologies could have other indirect effects. If the concentration of atmospheric carbon dioxide is reduced, CO_2_ could be released from the oceans [100]. As this CO_2_ in the ocean is driving ocean acidification, with damaging impacts on biodiversity [101], ocean outgassing of CO_2_ could relieve some ocean acidification, providing an indirect benefit for ocean biodiversity. Table 3 summarizes the likely biodiversity impacts of widespread implementation of greenhouse gas removal technologies.

**Table 3.** Summary of the likely biodiversity impacts of widespread implementation of land-based greenhouse gas removal technologies.

technology type	greenhouse gas removal technology	impact on processes affecting biodiversity	local biodiversity impact	catchment- scale biodiversity impact	regional/ global biodiversity impact
greenhouse gas removal	direct air capture	land footprint	0	0	0
greenhouse gas removal	enhanced mineral weathering	local impact of mining mineral/impact of spreading on land	−/0	−/0	0
greenhouse gas removal	soil carbon sequestration	enhancing soil organic matter stocks	+/0	+/0	+/0
greenhouse gas removal	biochar	providing microsites for soil microbiota	+	+	+
greenhouse gas removal	BECCS	large land footprint; direct and indirect effects	−	−	−
greenhouse gas removal	afforestation/ reforestation	large land footprint; direct and indirect effects	+/−	+/−	+/−
Earth system feedback	outgassing of CO_2_ from the ocean	de-acidification of the oceans	+/0	n.a.	+

While most mitigation measures listed in §3a tend to have positive local biodiversity impacts, greenhouse gas removal technologies are more mixed. BECCS will almost certainly lead to biodiversity conflicts if implemented at scales necessary to achieve greenhouse gas removals of approximately 10 Gt CO_2_, which would require 380–700 Mha of land [1]. In terms of impacts on biodiversity, soil carbon sequestration is neutral to positive, direct air capture neutral, and indirect effects of all greenhouse gas removal on ocean biodiversity might be positive; but for the others, evidence is mixed or, more often, the outcome is context-specific. For enhanced weathering, effects of spreading minerals are neutral, but mining will have an adverse local biodiversity impact. Afforestation/reforestation will depend on what tree species/mix are planted and what land use the new woodland replaces, but there are usually options to benefit biodiversity by woodland establishment [89]. In terms of mitigation–adaptation interactions, improving soil carbon sequestration and afforestation/reforestation are likely to show significant co-benefits, while BECCS, if implemented at scale, would probably yield some trade-offs [1,33].

## 4. Conclusion

Holding warming to 1.5°C versus 2°C can reduce significantly, the number of species facing a potential loss of 50% of their climatic range. Furthermore, there would be an increase of between 5.5% and 14% of the amount of the globe that could potentially act as climatic refugia for plants and animals. This is the equivalent, in terms of km^2^, to the current global protected area network [19]. From the additional analysis here, we further conclude that efforts to meet the 1.5°C target through mitigation efforts in the land sector would largely be consistent with biodiversity protection/enhancement depending on the mitigation approach used. Additional effort to meet the 1.5°C target using some greenhouse gas removal technologies (e.g. soil carbon sequestration) would be neutral or positive, whereas others are likely to lead to biodiversity conflicts (e.g. BECCS) when applied at scales necessary for meaningful greenhouse gas removal. However, if greenhouse gas removal technologies are used to manage an overshoot of emissions/temperature increase, there could be additional direct impacts on biodiversity compared to those that do not result in overshoot [3], because temperature will be higher than 1.5°C for a period of time, so scenarios that avoid overshoot would lead to fewer adverse impacts than those that result in overshoot. Many of the areas previously identified as showing high potential for some bioenergy crops directly overlap many of the areas of climatic refugia for many biodiversity taxa. Other land-based greenhouse gas removal options, such as afforestation/reforestation, are context-specific, but there is enough knowledge to implement these options in a manner that protects or enhances biodiversity, potentially offering adaptation benefits. Additional effort to meet the 1.5°C target, therefore, does present some risks, particularly if inappropriately managed, but it also presents some opportunities. More spatially explicit research is needed to look at the overlap between bioenergy, food security and biodiversity conservation.

Article 2 of the United Nations Framework Convention on Climate Change (UNFCCC) states that greenhouse gas concentrations be stabilized at … ‘a level that would prevent dangerous anthropogenic (human induced) interference with the climate system’ and ‘Such a level should be achieved within a time frame sufficient to allow ecosystems to adapt naturally to climate change, to ensure that food production is not threatened and to enable economic development to proceed in a sustainable manner’. This will require careful planning and cooperation that simultaneously deals with mitigation, energy, food, biodiversity and sustainable development—a point recognized in the spread of the Sustainable Development Goals. Thus, efforts must be taken to ensure that mitigation of greenhouse gas emissions is Article 2-compliant.

## Data accessibility

Wallace Initiative data can be accessed at http://Wallaceinitiative.org.
